# Characterizing the avian gut microbiota: membership, driving influences, and potential function

**DOI:** 10.3389/fmicb.2014.00223

**Published:** 2014-05-16

**Authors:** David W. Waite, Michael W. Taylor

**Affiliations:** Centre for Microbial Innovation, School of Biological Sciences, Faculty of Science, The University of AucklandAuckland, New Zealand

**Keywords:** bird, avian, microbiota, bacteria, 16S rRNA gene, meta-analysis

## Abstract

Birds represent a diverse and evolutionarily successful lineage, occupying a wide range of niches throughout the world. Like all vertebrates, avians harbor diverse communities of microorganisms within their guts, which collectively fulfill important roles in providing the host with nutrition and protection from pathogens. Although many studies have investigated the role of particular microbes in the guts of avian species, there has been no attempt to unify the results of previous, sequence-based studies to examine the factors that shape the avian gut microbiota as a whole. In this study, we present the first meta-analysis of the avian gut microbiota, using 16S rRNA gene sequences obtained from a range of publicly available clone-library and amplicon pyrosequencing data. We investigate community membership and structure, as well as probe the roles of some of the key biological factors that influence the gut microbiota of other vertebrates, such as host phylogeny, location within the gut, diet, and association with humans. Our results indicate that, across avian studies, the microbiota demonstrates a similar phylum-level composition to that of mammals. Host bird species is the most important factor in determining community composition, although sampling site, diet, and captivity status also contribute. These analyses provide a first integrated look at the composition of the avian microbiota, and serve as a foundation for future studies in this area.

## Introduction

The role of the gut microbiota in shaping the health and physiology of vertebrate hosts is a well-established, highly exciting area in microbiology. The diversity and function of microbes in the gastrointestinal (GI) tract is an area of ongoing research, with recognized roles for the vertebrate microbiota in nutrition (Jin et al., 1998; Preest et al., 2003; Turnbaugh et al., 2006; Angelakis and Raoult, 2010; Stanley et al., 2012), gut development (Stappenbeck et al., 2002; Rahimi et al., 2009; Zhang et al., 2011; Cao et al., 2012) and regulation of host physiology (Bäckhed et al., 2004; Björkholm et al., 2009; Meinl et al., 2009). 16S rRNA gene sequencing has been employed in a range of studies to assess the diversity and phylogenetic relationships of gut microbes and this has proven to be a powerful tool for understanding the factors that shape microbial communities, due to both its informative and predictive potential. A secondary benefit of the 16S rRNA gene is that, in addition to reporting the results of findings in scientific journals, it is customary to deposit the primary sequence data into publicly available databases which allow for a second wave of meta-study. By aggregating data from a variety of sources or environments, researchers have been able to discern large-scale patterns in microbial ecology, analysing the bacterial communities of mammalian (Ley et al., 2008a) and fish (Sullam et al., 2012) guts, as well as across other non-biological factors (Lozupone and Knight, 2007; Chu et al., 2010; Shade et al., 2013). One area that has arguably not undergone such a revolution is that of the avian microbiota. While several notable exceptions exist, such as commercially farmed broiler chickens and turkeys as well as the South American hoatzin, the majority of avian systems have not been studied outside of immediate pathogenic concerns.

Similar to other vertebrates, the GI tract of birds is colonized by a community of microbes, with a density as high as 10^11^ c.f.u/g in the hindgut (Barnes, 1972). The role of microbes in the avian gut has long been a topic of study, with ground-breaking research throughout the 1960's identifying the role of bacteria in starch degradation and volatile fatty-acid production within the bird gut (Bolton, 1965; Annison et al., 1968; Pritchard, 1972). From a microbiological perspective, there are two major areas of interest in the bird gut. The crop, a muscular pouch located at the start of the alimentary tract, is associated with the breakdown of starch (Shaw, 1913; Pritchard, 1972; Vispo and Karasov, 1997; Pacheco et al., 2004), and microbially mediated fermentation of lactate (Bolton, 1962, 1965; Pritchard, 1972; Moore et al., 2004). Cellulolytic microbes have occasionally been observed in avian crops (Shetty et al., 1990; Domínguez-Bello et al., 1993), but significant bacterial cellulolysis has only been reported in the hoatzin (Grajal et al., 1989; Domínguez-Bello et al., 1993), with only low levels of cellulose fermentation reported for other birds (Clemens et al., 1975; Cutler et al., 2005). The ceca are the sites of recycling of urea (Barnes, 1972; Mead, 1989; Vispo and Karasov, 1997; Preest et al., 2003), retention of water (McNab, 1973) and fermentation of carbohydrates (Józefiak et al., 2004). It has been observed that a cellulose-rich diet leads to increased size of the ceca (Leopold, 1953; McNab, 1973; Miller, 1976; Duke et al., 1984; Redig, 1989; Stevens and Hume, 1998), but there is contradictory evidence for the direct utilization of cellulose in the avian hindgut (Barnes, 1972; McNab, 1973; Mead, 1989).

With the rise of 16S rRNA gene sequencing a large portion of avian microbiology has shifted from microbial physiology to the diversity and phylogeny of avian gut microbes. Specific studies have addressed areas of avian microbial ecology, such as the variation in microbial diversity along the GI tract (Bjerrum et al., 2006; Gong et al., 2007; Torok et al., 2008; Waite et al., 2012), the influence of diet (Rubio et al., 1998; Blanco et al., 2006; Torok et al., 2008; Janczyk et al., 2009; Hammons et al., 2010), age (Van Der Wielen et al., 2002; Godoy-Vitorino et al., 2010; Van Dongen et al., 2013) or other host-specific factors (Zhu et al., 2002; Lucas and Heeb, 2005; Banks et al., 2009; Benskin et al., 2010; Wienemann et al., 2011). While there is extensive evidence that microbial colonization of the GI tract brings benefits to the host bird (Jin et al., 1998; Torok et al., 2008; Angelakis and Raoult, 2010; Torok et al., 2011; Zhang et al., 2011; Cao et al., 2012; Stanley et al., 2012), there are also pathways through which the normal colonization of microbes can be of detriment to the host (Ford and Coates, 1971; Potti et al., 2002; Cao et al., 2012; Singh et al., 2013). Although there are many published studies exploring aspects of the avian microbiota, it has evidently been uncommon for authors to publish their sequence data to an archive, somewhat limiting the potential for avian metastudies. As an example of this, in their 2008 meta-analysis of the vertebrate microbiota Ley et al. had access to rich clone-library data from insects (19 studies), humans (20 studies) and other vertebrate species (23 studies, including five from birds) (Ley et al., 2008b). In 2012, Sullam et al. identified for analysis 24 pre-existing clone-libraries derived from fish guts (Sullam et al., 2012). By contrast, in the same year Kohl only identified eight avian libraries with any significant microbiota data (Kohl, 2012). A survey of the recent literature has shown that the picture of the avian microbiota has since improved significantly, with the continued usage of clone-libraries and incorporation of amplicon pyrosequencing into existing study systems (Table 1).

**Table 1:** **Published sequence data obtained from molecular analysis of avian samples**.

**16S rRNA gene clone data**			**16S rRNA gene amplicon data**				
**Host**	**Site sampled**	**References**	**Source**	**Host**	**Site sampled**	**Data ID**	**References**
Adelie penguin	Faecal	Banks et al., 2009	MG-RAST	Turkey	Ileum	4514500.3–4514537.3	Danzeisen et al., 2013
Capercaillie	Cecum	Wienemann et al., 2011		Chicken	Cecum	4537568.3–4537604.3	Stanley et al., 2013
Chicken	Cecum	Zhu et al., 2002	NCBI SRA	Chicken, duck, goose	Faecal	PRJEB2135	Unno et al., 2010
	Illeum/Cecum	Lu et al., 2003		Chicken	Cecum	PRJNA193217	Unknown
	Cecum	Bjerrum et al., 2006			Ileum	PRJEB1467	Unknown
	Crop/Cecum	Gong et al., 2007			Faecal	PRJNA169064	Unknown
	Cecum	Torok et al., 2011		Emu	Cecum	PRJNA194064	Bennett et al., 2013
	Aggregate	Wei et al., 2013		Kakapo	Crop/Faecal	PRJNA222380	Waite, unpublished
Crane	Faecal	Ryu et al., 2012		Little blue penguin	Cloaca	PRJEB3384	Unknown
Hoatzin	Crop	Godoy-Vitorino et al., 2008		Misc. penguins^*^	Faecal	PRJEB3083	Dewar et al., 2013
	Crop	Wright et al., 2009		Petrel/Prion^*^	Faecal	PRJEB1549	Unknown
	Crop	Godoy-Vitorino et al., 2010					
Kakapo	Crop/Faecal	Waite et al., 2012					
Shorebirds^*^	Cloaca	Santos et al., 2012					
Gull	Faecal	Lu et al., 2008					
Parrot^*^	Cloaca	Xenoulis et al., 2010					
Ostrich	Cecum	Matsui et al., 2010					
Stork	Feathers	Nawrot et al., 2009					
Turkey	Cecum	Scupham, 2007					
	Faecal	Lu and Domingo, 2008					
	Cecum	Scupham et al., 2008					
	Aggregate	Wei et al., 2013					

Asterisk (

* *) denotes a study that analyzed the bacterial communities associated with multiple species of birds, but with common phylogenetic or geographic grouping. For 16S rRNA gene amplicon data, reference names are the last name of submitter where available. Short-read data with an unknown reference refers to data which could not be tracked back to a published paper*.

In order to gain new insights into the avian gut microbiota, we sought to amalgamate the existing knowledge and determine whether patterns detected in individual studies were consistent across avians as a whole. To achieve this goal we collected publicly available data from NCBI GenBank and MG-RAST and reanalyzed the data using established bioinformatics pipelines.

## Methods

### Data acquisition and quality control

Clone-library data were obtained from GenBank through a comprehensive literature survey, followed by the retrieval of clone-library sequence data of interest. Short amplicon data from next-generation sequencing studies were obtained from MG-RAST and the NCBI Sequence Read Archive (hereafter referred to as short-read data) by browsing for the publicly available data sets. Data sources are as reported in Table 1, with the exception of the database provided by Wei and colleagues (Wei et al., 2013), which was excluded from analysis as their data overlapped significantly with sequences obtained from original studies.

All downloaded data were re-analyzed using mothur version 1.32.1 (Schloss et al., 2009). For short-read data, flowgrams were trimmed to a single length then denoised. Where flowgrams were not available, sequences were trimmed using the trim.seqs command, removing the barcode and primer sequences and discarding sequences with an average quality score of less than 25, or sequences with a homopolymer run of greater than eight bases. All sequence data were then aligned, screened for chimeras with uchime (Edgar et al., 2011) and classified against the Greengenes taxonomy using the naïve Bayesian method (Desantis et al., 2006; Wang et al., 2007). Sequences that could not be classified to domain level, or were classified as *Cyanobacteria*, were removed from the dataset as they likely represent ingested plant material. Chimeric sequences and sequences that could not be aligned were also removed from the data set.

For data obtained from clone libraries it is common practice to simply upload representative sequences to GenBank, rather than the complete dataset. In order to account for the loss of abundance information from the original clone libraries, taxonomic classification was reported by calculating operational taxonomic units (OTUs) of 97% sequence similarity for each sample and assigning taxonomy using the classify.otu command in mothur. Although short-read data does contain the data from the complete sequencing run, studies did not always utilize the same 16S rRNA gene region and so could not be directly compared. In lieu of OTU generation, genus-level phylotypes were constructed using the sequence classification. For short-read data, the phylotype table was rarefied to a depth of 1500 data points and Shannon and Simpson diversity indices calculated.

### Correlating metadata to community structure

For clone data, sequences were trimmed to an 800 bp overlapping region and a phylogenetic tree constructed using the clearcut neighbor-joining algorithm (Evans et al., 2006) for UniFrac analysis. Sequences less than 800 bp in length were discarded, resulting in the loss of three avian samples compared with the previous classification. Due to the potential bias in relative abundance incurred by the selective uploading of data, only unweighted UniFrac distance was calculated. For short-read data there was no contiguous region of sequence common to all samples, so analysis was performed by constructing genus-level phylotypes of the classified data. Community differences were calculated using Jaccard (presence/absence) and Yue-Clayton theta (abundance) distance by randomly subsampling each community to 1500 sequences 20,000 times and averaging the community distances across iterations.

Metadata regarding the host, sample type, animal diet and captivity status were recorded and their impact on community differences compared using the vegan package (version 2.0–8) (Oksanen et al., 2013) in the R software environment (R. Core Team, 2012). Samples were grouped according to the following categories: host animal, diet and captivity status. Diet consisted of three categories—carnivore, herbivore and grain-fed—that reflected a “typical” diet of the host. When dividing animals based on diet, the distinction was made between an herbivorous diet (leaves and green plant material, such as eaten by the kakapo and hoatzin) and grain-fed diet (pelleted feed, such as found in farmed chickens) due to the different nutrient content and availability in these diets. Captivity status consisted of simply dividing samples into those animals that are wild or farm-raised. For short-read data the study that provided the data was also used as a test for how much the dynamics of the study itself shaped the data. This factor could not be applied to the clone-library data as not every original study uploaded sequences with sufficient information to recapture biological replication with the sequence data.

Permutational multivariate analysis of variance (PERMANOVA) with linear model fitting was performed (Anderson, 2001; McArdle and Anderson, 2001) in R. Samples were grouped according to each metadata factor and tested for how well the grouping accounted for the variation between samples using the “Adonis” function of the vegan package (Oksanen et al., 2013), measured as *R*^2^. A significance value (*p*-value) was generated by comparing the obtained *R*^2^ to that obtained from 1000 random permutations of the data. For factors with a statistically significant fit, constrained canonical analysis (CCA) was performed (Ter Braak, 1986) using the factor as the constraining variable to isolate the contribution of that factor to the microbial community.

### Functional prediction of gut microbiota

Following quality control of short-read data, sequences were mapped to OTUs using closed-reference OTU picking in QIIME 1.80 (Caporaso et al., 2010). 16S rRNA gene abundance levels were then normalized against the known gene copy number for that OTU and function predictions made based on OTU membership using PICRUSt (Langille et al., 2013). Functional predictions were categorized into KEGG pathways and statistical analysis performed using STAMP v2.0 (Parks and Beiko, 2010). Data were partitioned by metadata factors and differences in relative abundance tested using ANOVA, followed by *post-hoc* Games-Howell test with the Benjamini-Hochberg FDR used as a multiple testing correction (Benjamini and Hochberg, 1995). For testing the presence of genes involved in cellulose digestion, KEGG data were screened for pathways that mapped to COGs involved in cellulolysis and data extracted. Pair-wise comparisons were performed using Welch's *t*-test (Welch, 1947) with the Benjamini-Hochberg FDR.

## Results and discussion

### Taxonomic classification of OTUs

**Figure 1:**
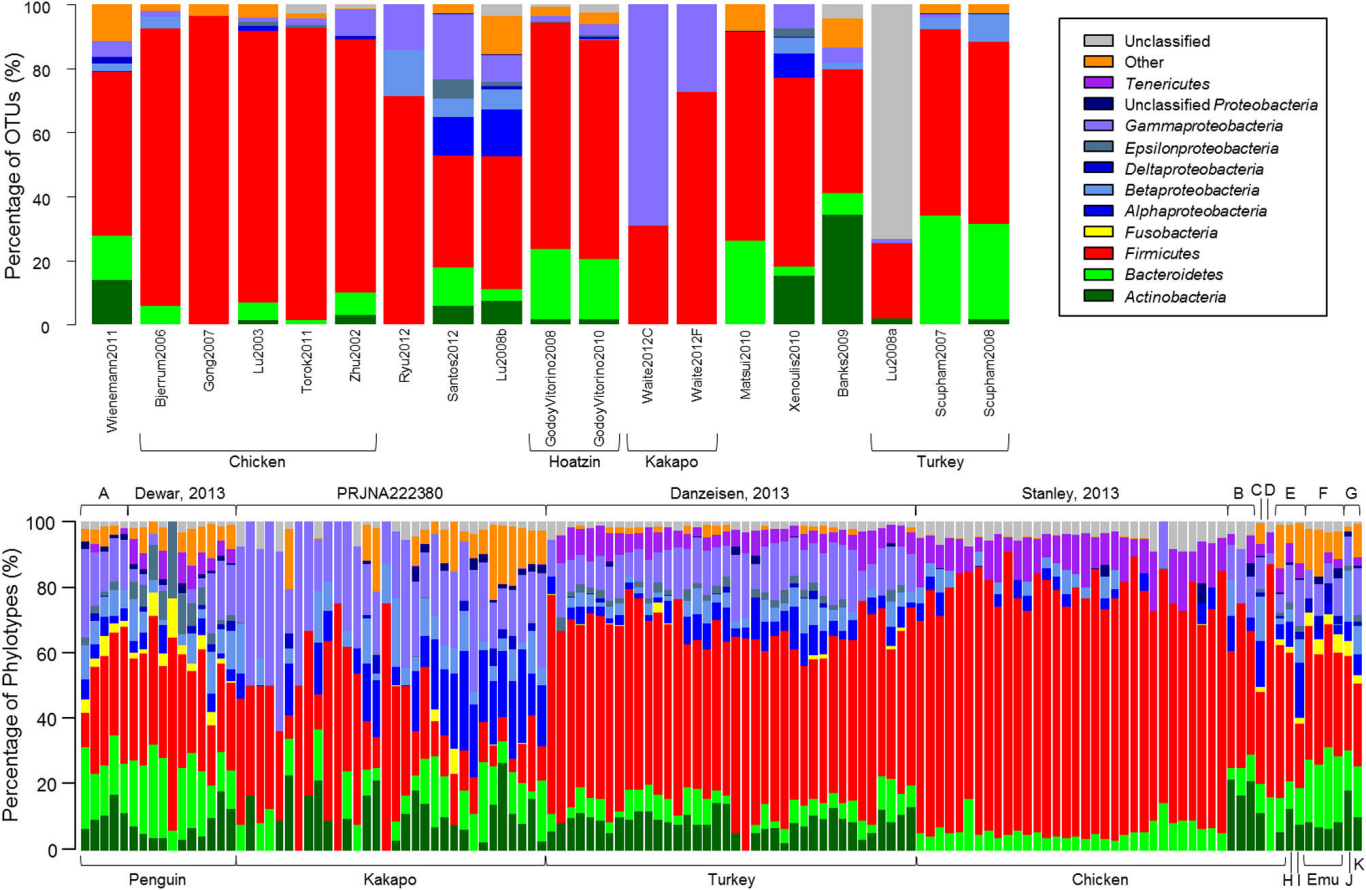
**The relative proportion of OTUs represented in each study**. OTUs were constructed by calculating average-neighbor distance between aligned 16S rRNA gene sequences in mothur and classified as a cluster of sequences with ≥97% similarity. Taxonomic classification for each OTU was derived from a consensus taxonomic classification of each sequence assigned to the OTU. **(Top)** Samples from clone-library data. **(Bottom)** Next-generation sequencing samples obtained from Sequence Read Archive. Top labels identify the study from which sequences were downloaded; bottom labels identify the host bird. Top letters denote studies PRJEB3384 (A), PRJEB1467 (B), PRJNA169064 (C) PRJNA193217 (D), Unno, 2010 (E), Bennet, 2013 (F), and PRJEB1549 (G). Bottom letters denote host organisms duck (H), goose (I), fairy prion (J), and petrel (K).

Quality-control of sequence data yielded a high number of high-quality sequences, of varying length, from a subset of the studies reported in Table 1, (Tables 2A,B). Consistent with the microbiota of vertebrates in general, the avian gut microbiota appears to harbor mostly OTUs belonging to *Bacteroidetes*, *Firmicutes*, and *Proteobacteria* (Figure 1). Members of the phylum *Firmicutes* were present in all samples analyzed, while *Proteobacteria* and *Bacteroidetes* were also widespread (*Proteobacteria*: 90% of clone samples, 100% of short-read samples; *Bacteroidetes*: 80% of clone samples, 87% of short-read samples). These three phyla are commonly observed within gut environments, and specific lineages of these phyla are frequently studied for their symbiotic roles, for example *Bacteroides thetaiotaomicron* starch degradation in humans (Dongowski et al., 2000; Xu et al., 2003; Sears, 2005), and *Lactobacilli*-associated bile salt hydrolase activity in mice and chickens (Tannock et al., 1989; Tanaka et al., 1999; Knarreborg et al., 2002). To a lesser extent, *Actinobacteria* (65% of clone samples, 89% short-read samples) and *Tenericutes* (65% of clone samples, 58% short-read samples) were also reasonably common throughout the data. Within the short-read data, a higher proportion of unclassified OTUs was observed, which may be due to a lack of phylogenetic resolution due to shorter read length. Alternatively, it has been shown that the use of the adapter/barcode construct in a single-step PCR, as is commonplace in pyrosequencing studies, can negatively affect taxonomic classification (Berry et al., 2011).

Table 2**Number of reads used, OTUs generated and average sequence length for 16S rRNA gene data utilized in the study**.

**Table:** 

**(A) Host**	**Site sampled**	**Number sequences**	**Number OTUs**	**Median sequence length (bp)**	**Figure 1A label**
Adelie penguin	Faecal	48	44	846	Banks, 2009
Capercaillie	Cecum	114	43	1476	Wienemann, 2011
Chicken	Cecum	329	213	433	Zhu, 2002
	Illeum/Cecum	99	72	644	Lu, 2003
	Cecum	74	52	1404	Bjerrum, 2006
	Crop/Cecum	39	27	850	Gong, 2007
	Cecum	627	137	301	Torok, 2011
Crane	Faecal	16	7	817	Ryu, 2012
Hoatzin	Crop	1235	376	1365	Godoy-Vitorino, 2008
	Crop	2123	267	1338	Godoy-Vitorino, 2010
Kakapo	Crop	29	6	728	Waite et al., 2012
	Faecal	73	17	740	Waite et al., 2012
Shorebirds^*^	Cloaca	64	34	192	Santos, 2012
Gull	Faecal	117	85	780	Lu, 2008b
Parrot^*^	Cloaca	49	39	684	Xenoulis, 2010
Ostrich	Cecum	310	98	889	Matsui, 2010
Turkey	Cecum	657	139	1450	Scupham, 2007
	Faecal	688	423	472	Lu, 2008a
	Cecum	104	67	1454	Scupham, 2008

**Table:** 

**(B) Host**	**Individuals sampled**	**Number of sequences**	**Region sequenced**	**Number of phylotypes**	**Median sequence length (bp)**	**Shannon diversity**	**Shannon evenness**	**Simpson diversity**	**Figure 1B label**
Turkey	38	910,992	~V3	60	160	1.27	0.17	0.48	Danzeisen, 2013
Duck	1	6742	V1–V3	105	481	1.73	0.24	0.33	Unno, 2010
Goose	1	7825	V1–V3	232	484	3.40	0.46	0.08	Unno, 2010
Chicken	1	6416	V1–V3	112	486	2.90	0.40	0.10	Unno, 2010
	32	74,678	V1–V2	20	515	1.60	0.22	0.30	Stanley, 2013
	3	16,990	~V2	24	195	0.56	0.08	0.72	PRJEB1467
	1	13,243	~V2	31	168	2.07	0.28	0.17	PRJNA193217
	1	22,384	~V3	204	154	3.37	0.46	0.08	PRJNA169064
Emu	4	96,549	~V2	39	219	1.44	0.20	0.34	Bennet, 2013
Kakapo	30	128,021	V3–V4	28	268	0.83	0.11	0.56	PRJNA222380
Little penguin	4	68,280	V2	53	188	0.86	0.12	0.56	PRJEB3384
King penguin	8	116,937	~V2	50	288	1.98	0.27	0.22	Dewar, 2013
Misc. penguins^*^	3	18,216	V1–V3	120	285	2.95	0.40	0.10	Dewar, 2013
Petrel/Prion	2	17,335	~V2	107	384	2.63	0.36	0.18	PRJEB1549

Asterisk (

* *) denotes a study that analyzed the bacterial communities associated with multiple species of birds, but with common phylogenetic or geographic grouping. (A) Data obtained from clone-library based studies and the published study that reported the sequences. (B) Data obtained from short-read studies. Note that phylotypes are used instead of OTUs due to differing gene regions being sequenced. Reported regions sequenced are only approximate and do not accurately reflect the start/stop positions of the amplicons. Ecological diversity estimators were calculated by rarefying phylotype table to 1500 phylotypes/sample prior to calculation and median values are reported. Shannon Evenness is calculated by dividing the Shannon Diversity by the maximum Shannon Diversity value for the depth of sampling. A value of 1 represents complete evenness*.

### Factors shaping the avian microbiota: study vs. host

PERMANOVA testing of the short-read data set revealed that the largest factor contributing to the shaping of the microbiota was the study itself (Table 3). This finding may be a real result, as most studies focused on a single bird geographically isolated from other studies (i.e., the “study” variable is the product of host and location), or may be an artefact resulting from the specific DNA extraction and PCR techniques involved (Boom et al., 1990; Suzuki and Giovannoni, 1996; Martin-Laurent et al., 2001; Sipos et al., 2007; Berry et al., 2011; Kennedy et al., 2014). In order to resolve this issue, we hypothesized that if the host species was truly driving the differences observed between studies, then the phylogenetic differences between taxonomically similar bacterial lineages within each study would be smaller between studies with a closely related host bird. Alternatively, a study that investigated a range of host birds would have greater within-study variation than a study that investigated a single host.

**Table 3:** **Calculated fit of metadata factors to community distances using PERMANOVA with linear model fitting**.

**Clone-library**	**Unweighted UniFrac**	**Fit (*R*^2^)**	**Significance**
	Host	0.68	0.001
	Sample site	0.25	0.001
	Diet	0.17	0.002
	Captivity	0.09	0.004
**Short-read amplicon**	**Jaccard Distance**		
	Study	0.40	0.001
	Host	0.35	0.001
	Sample site	0.27	0.001
	Diet	0.18	0.001
	Captivity	0.13	0.001
**Short-read amplicon**	**Yue-Clayton theta**		
	Study	0.41	0.001
	Host	0.36	0.001
	Sample site	0.31	0.001
	Diet	0.21	0.001
	Captivity	0.15	0.001

*For both data types the sample collection method was tested but did not show any meaningful correlation with community structure*.

**Figure 2:**
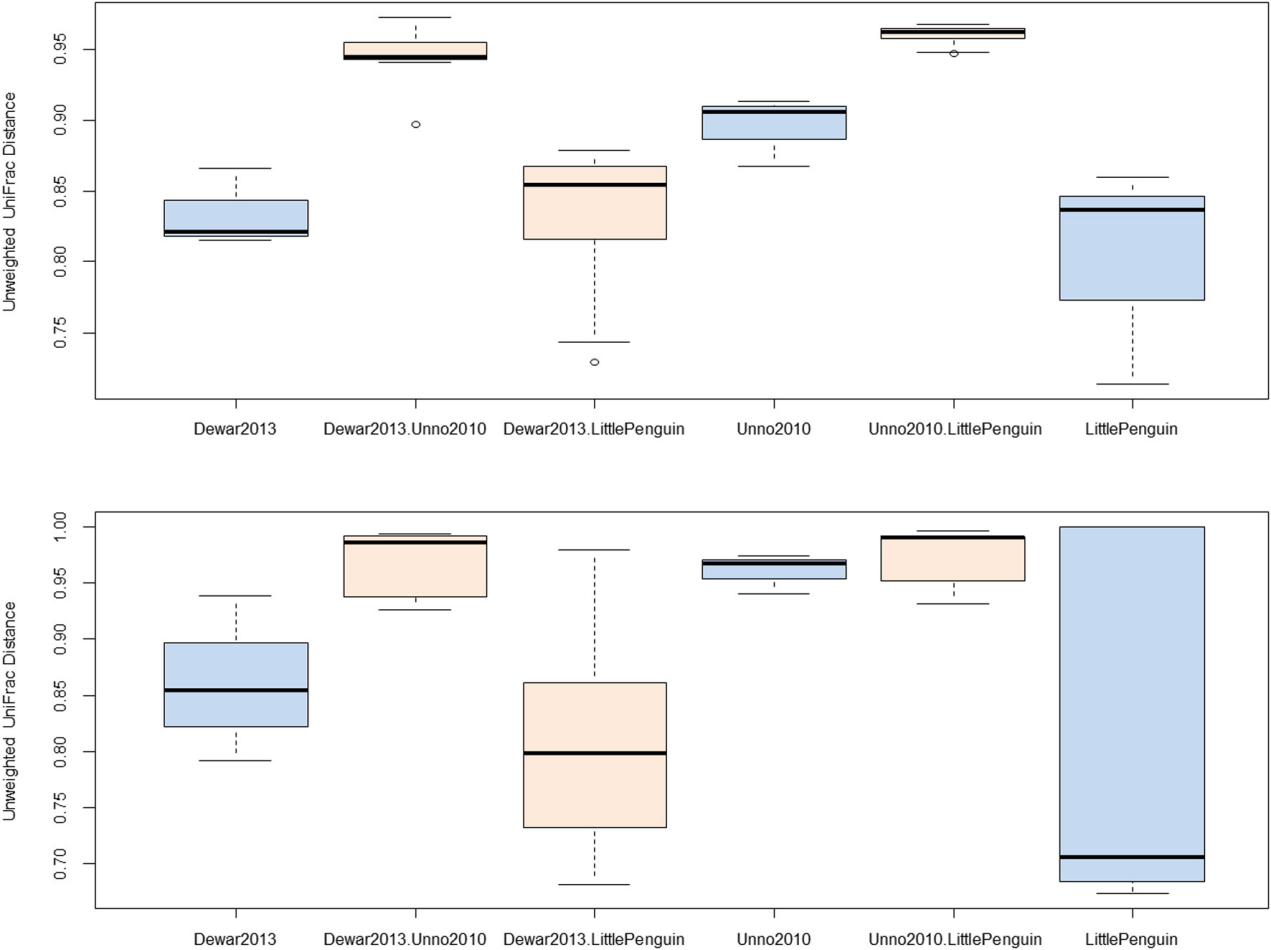
**Unweighted UniFrac distances for within- and between-study comparisons**. Distances were calculated by extracting reads classified as *Clostridium* (top) and *Bacteroidetes* (bottom) from each sample and constructing neighbor-joining phylogenetic trees based on average-neighbor distances between aligned sequences. Differences between each pair of samples were categorized as being the distance between samples from the same study or from different studies and plotted accordingly (blue = within study, orange = between study). The study “Dewar, 2013” investigated the faecal microbiota from little, king, macaroni, and gentoo penguins. The study “LittlePenguin” investigated the faecal microbiota of little penguins, and “Unno, 2010” the microbiota of a chicken, duck, and goose from a farm.

We identified three studies that sequenced overlapping regions of the bacterial 16S rRNA gene (Table 1, Unno, 2010, Dewar, 2013 and PRJEB3384) and observed that two bacterial genera were conserved across all three studies, namely *Bacteroides* and *Clostridium*. Sequences associated with these taxa were extracted from the main dataset and unweighted UniFrac distances were calculated between each biological replicate. The within- and between-study UniFrac distances are reported in Figure 2 and, consistent with our prediction, the within-study and between-study difference was similar when the data originated from a closely related host (Figure 2, Dewar, 2013, LittlePenguin and Dewar, 2013. LittlePenguin). By contrast, the differences between Dewar, 2013 and Unno, 2010, and LittlePenguin and Unno, 2010, were higher than the within-group difference for *Clostridium* and elevated compared to the penguin/penguin comparisons for *Bacteroides*. The within-group differences were higher for Unno, 2010-*Bacteroides* than for other groups, but this may be a result of the Unno, 2010 study itself analysing several different birds. Although the different methodologies employed in the various studies are likely to have some impact on the results, we concluded that this was overshadowed by the impact of the host organism and proceeded to analyse other metadata factors.

### Factors shaping the avian microbiota: biological factors

**Figure 3:**
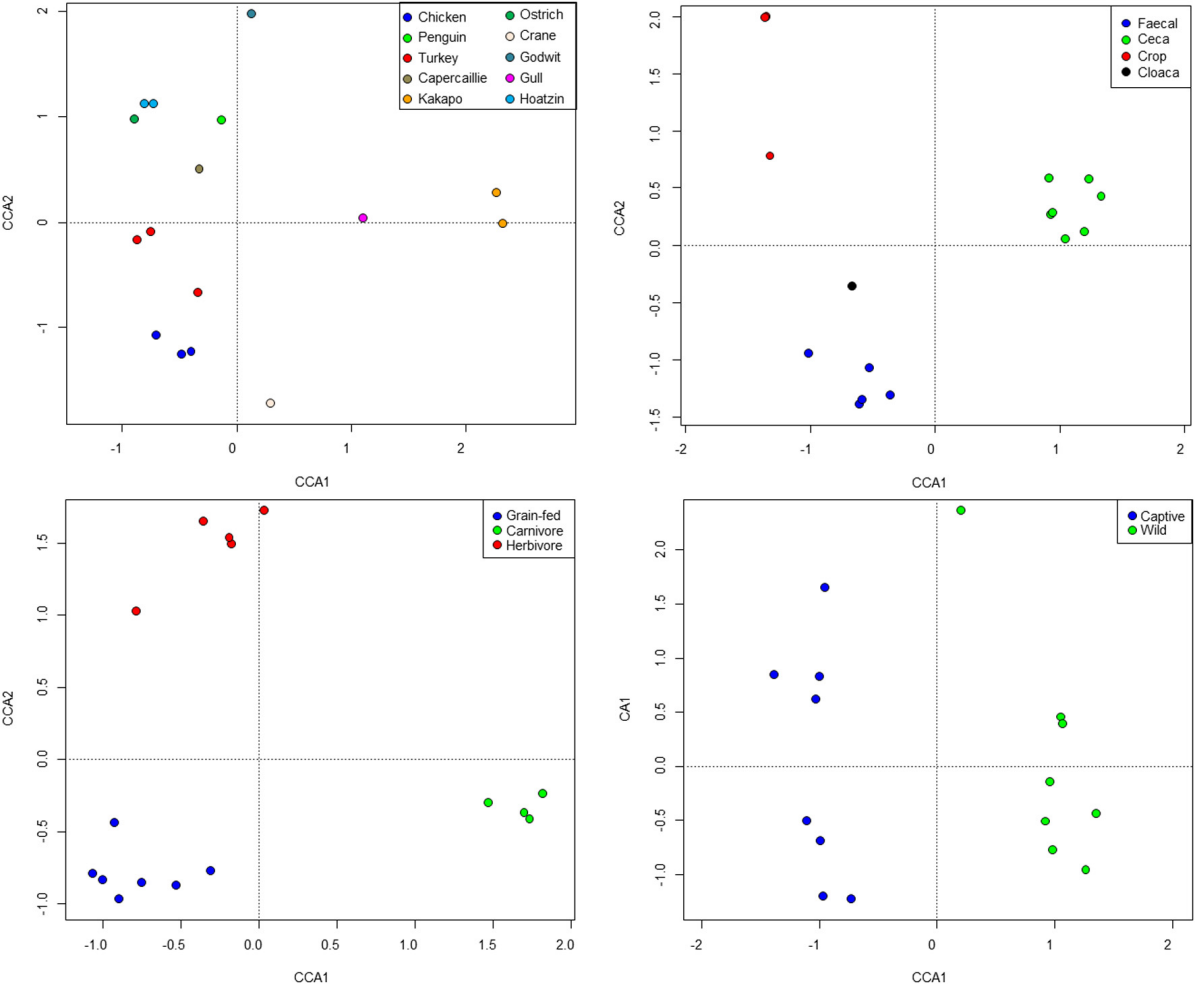
**Constrained Canonical Analysis of community structure based on fitting of metadata factors to the clone-library sequence data**. Images represent host (top left), sample site (top right), diet (bottom left), and captivity status (bottom right).

**Figure 4:**
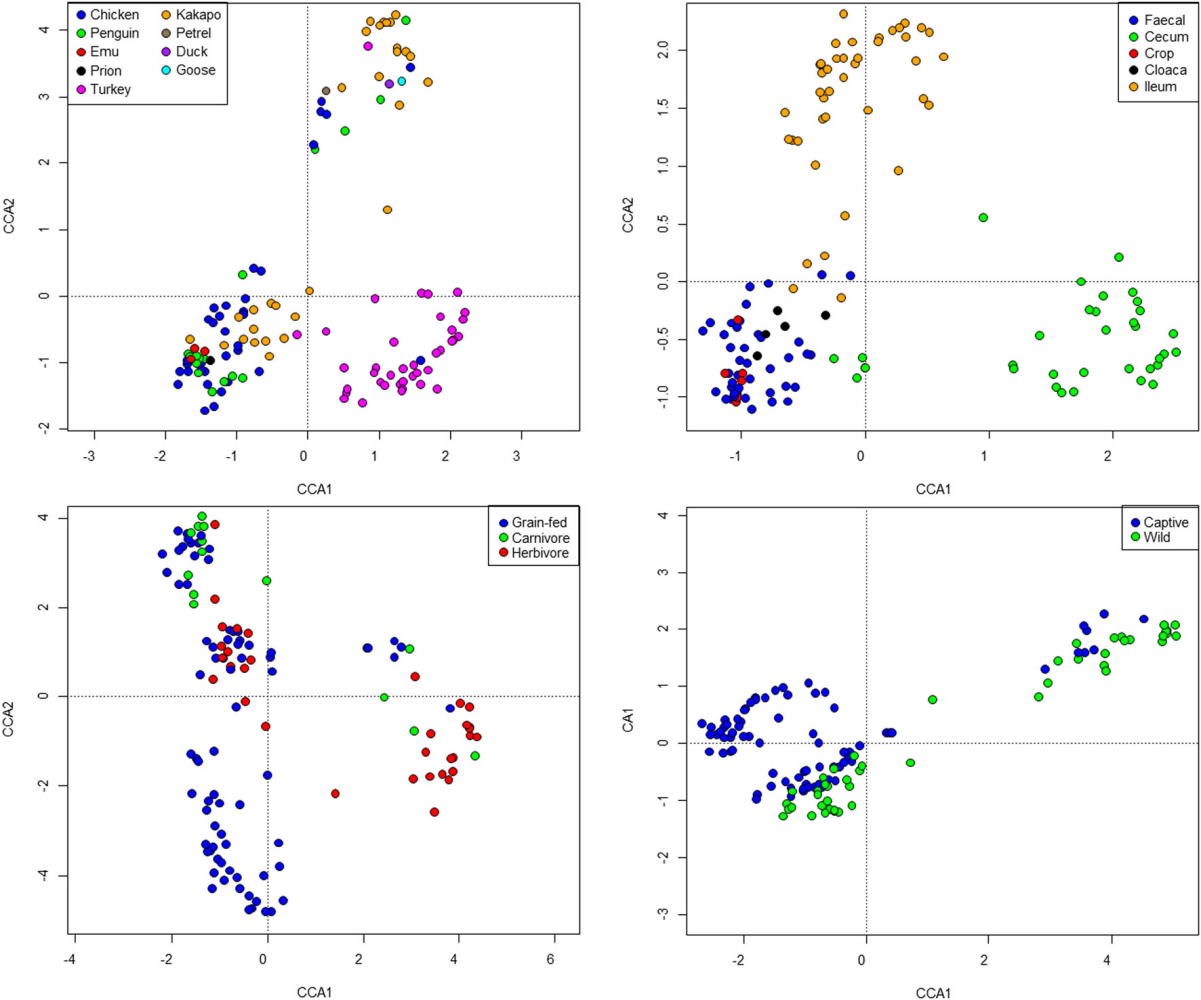
**Constrained Canonical Analysis of community structure based on fitting of metadata factors to the short-read sequence data**. Images represent host (top left), sample site (top right), diet (bottom left), and captivity status (bottom right). Note that the “herbivore” grouping represents exclusively kakapo.

Standard ecological diversity indices revealed varying degrees of microbial diversity among the birds studied (Table 2B). In agreement with our previous observations of low microbial diversity within the kakapo hindgut (Waite et al., 2012, 2013), the diversity estimators for kakapo were among the lowest observed. Consistent with previously reported mammalian findings (Ley et al., 2008a), and with more targeted avian studies (Zhu et al., 2002; Lucas and Heeb, 2005; Banks et al., 2009; Benskin et al., 2010), the host organism was the strongest driver of community structure in the clone-library data and second strongest in the short-read data (Table 3). Other factors were still significantly associated with shaping the gut community but their fit to the data was lower. The fit for any particular factor across the data was quite low (Table 3), which is likely a result of compounding variables from the individual studies, rather than a real lack of influence of these factors. In order to account for this variation, CCA was used to visualize patterns in the data that could be accounted for by the factor of interest. Results are summarized in Figure 3 and show clear clustering of data for clone samples, but weak clustering for short-read data (Figure 4). This lack of resolution within the short-read data is likely due to the loss of OTU phylogenetic information due to non-overlapping 16S rRNA gene regions between studies. Due to the lack of phylogenetic relationship between OTUs, each OTU is considered equally different from every other OTU (Lozupone and Knight, 2005) and hence evolutionary information is lost.

### Functional prediction of the gut microbiota

Ultimately, the study of microbial communities is of little biological value unless the functional potential of the community, or individual members, is considered. Statistical testing revealed differences in many predicted functional pathways when data were partitioned by host, but this finding was ignored as it is a likely side-effect of 16S rRNA prediction (i.e., if the 16S rRNA-defined communities differ between hosts, the metagenomic prediction based on 16S rRNA community is also likely to differ). Metagenomes were instead partitioned by diet, captivity and gut location sampled and these categorizations of data revealed interesting differences in functional capability (Table 4). Captive birds were predicted to have a microbiota with enhanced capability for carbohydrate metabolism and a lower rate of microbial genes associated with infectious disease. When comparing predicted metagenomes by diet, the microbiota of carnivores was predicted to have a greater capability for amino acid and energy metabolism when compared to herbivores, a finding previously reported in mammals (Muegge et al., 2011). The grain-fed microbiota was predicted to have a higher capability for carbohydrate metabolism than that of herbivores. Genes involved in lactate production were predicted in all samples, which is not surprising as lactate is a known by-product of microbial activity in the ceca and is a major metabolic precursor for glucose in avians (Brady et al., 1979; Ogata et al., 1982; Franson et al., 1985). These findings provide support for the fitting of metadata categories to the samples, as the factors that contribute to shaping the microbiota were also supported by known functional roles of these microorganisms. Partitioning of data by sample site revealed several key influences on the predicted functionality of the microbiota. For example, genes grouping into the KEGG grouping “signaling molecules and interaction” were lowest in faecal samples. This grouping includes an array of genes involved in cell adhesion molecules and cytokine receptors and is likely to be involved in host/bacteria interactions. Genes involved in carbohydrate metabolism were at their lowest in foregut samples from kakapo, and elevated in the hindgut, consistent with the fact that most birds utilize their hindgut/cecum for carbohydrate fermentation (McNab, 1973; Mead, 1989).

**Table 4:** **Summary of key findings in differences between predicted metagenomes**.

**Functional group**	**Sample 1**	**Proportion of metagenome (%)**	**Sample 2**	**Proportion of metagenome (%)**	***p*-value (corrected)**
Carbohydrate metabolism	Captive	11.28	Wild	10.49	<0.001
	Grain-fed	11.51	Carnivore	10.85	<0.001
			Herbivore	10.68	<0.001
Infectious disease	Wild	0.50	Captive	0.43	0.002
Amino acid metabolism	Carnivore	10.86	Herbivore	8.52	<0.001
			Grain-fed	8.81	0.026
Signaling molecules and interaction	Faecal	0.16	Crop	0.20	0.017
			Cecum	0.25	0.006
			Ileum	0.23	<0.001
β-1,4-endoxylanase	Carnivore	0.019	Herbivore	0.008	0.01
β-xylosidase	Grain-fed	0.015	Herbivore	0.007	0.001
Xylanase	Herbivore	0.007	Grain-fed	0.002	<0.001

*Comparisons are reported as column Sample 2 compared to the last entry in Sample 1. Gene abundances are reported as a relative proportion of the total predicted metagenomic content*.

Interestingly, the influence of diet did not match differences in the predicted ability of the microbiota to degrade cellulose. Between the three diet groupings, β-1,4-endoxylanase was more abundant in carnivorous birds than herbivorous birds. β-xylosidase activity was predicted to be higher in grain-fed birds than strictly herbivorous birds, while xylanase was higher in herbivorous birds than grain-fed (Table 4). When taken as a proportion of the total cellulolytic potential, the microbiota of carnivorous birds had a higher predicted occurrence of β-xylosidase than that of herbivorous birds, and a higher occurrence of Cellulase M than grain-fed birds. Between the non-carnivorous birds, Cellulase M and xylanase accounted for a higher proportion of cellulolytic potential in the herbivorous birds, and β-glucosidase and β-xylosidase in grain-fed birds. These genes were detected in a range of bacterial phyla within the avian gut, but particular bacterial families were enriched in the gut microbiota, likely contributing to these differences in relative gene abundance. Of the PICRUSt OTUs that carried cellulolytic potential, members of the *Bifidobacteriaceae*, *Bacteroidaceae*, and *Lactobacillaceae* were highly represented in metagenomes which exhibited elevated β-xylosidase and β-glucosidase levels. *Leuconostocaceae* were enriched in predicted metagenomes with elevated Cellulase M and β-xylosidase. Interestingly, higher abundance of xylanase genes was pre-dominantly associated with abundance of the *Enterobacteriaceae*, which may reflect the influence of the *Proteobacteria*-rich kakapo microbiota. When normalized to a proportion of the total cellulolytic gene abundance, predicted proportions of β-1,4-endoxylanase were not significantly different between dietary groupings. Although not necessarily intuitive, these findings are supported by previous observations that the cellulolytic potential of the avian hindgut is minimal (Barnes, 1972; McNab, 1973; Mead, 1989), and correlates with the observation that cellulolytic pre-digestion of feed boosts energy harvest and weight gain (Józefiak et al., 2006; Yu et al., 2008; Cowieson et al., 2010; Mathlouthi et al., 2011; Ghahri et al., 2012; Ribeiro et al., 2012) in farmed broiler chickens. Caution must be taken in interpreting these predictions, as a recent study has shown that the functional capabilities of the gut microbiota are dependent on community membership as well as genetic potential (Berry et al., 2013). Furthermore, the PICRUSt prediction framework can only account for sequences that can be accurately mapped to the existing database, with no provision for sequences representing novel, or unstudied, bacterial lineages. Nevertheless, the framework provided high-level predictions that were consistent with the known state of avian microbiology and therefore represents an excellent pathway for generation of novel hypotheses and for general annotation of 16S rRNA gene amplicon studies.

In summary, we have conducted a comprehensive meta-analysis of publicly available avian microbiota sequences and tested whether, despite notable differences in physiology between avians and mammals, the factors that drive community structure are the same. We show that the avian host species is the strongest factor in determining community composition and decoupled this effect from potential study bias where the data allowed. Finally, we have analyzed the potential functional profiles of 16S rRNA gene amplicon data and found that the genomic potential predicted of the communities fits well with the existing literature, and is therefore an excellent platform to leverage these data into new hypotheses and lines of inquiry.

### Conflict of interest statement

The authors declare that the research was conducted in the absence of any commercial or financial relationships that could be construed as a potential conflict of interest.
